# In Silico Mining for Antimalarial Structure-Activity Knowledge and Discovery of Novel Antimalarial Curcuminoids

**DOI:** 10.3390/molecules21070853

**Published:** 2016-06-29

**Authors:** Birgit Viira, Thibault Gendron, Don Antoine Lanfranchi, Sandrine Cojean, Dragos Horvath, Gilles Marcou, Alexandre Varnek, Louis Maes, Uko Maran, Philippe M. Loiseau, Elisabeth Davioud-Charvet

**Affiliations:** 1 Institute of Chemistry, University of Tartu, 50411 Tartu, Estonia; birgit.viira@gmail.com (B.V.); uko.maran@ut.ee (U.M.); 2 Bioorganic and Medicinal Chemistry Team, UMR 7509 CNRS-Université de Strasbourg, European School of Chemistry, Polymers and Materials (ECPM), 25, rue Becquerel, Strasbourg F-67087, France; t.gendron@ucl.ac.uk (T.G.); don.antoine.lanfranchi@gmail.com (D.A.L.); 3 Laboratoire de Chemoinformatique, UMR7140 CNRS-Université de Strasbourg, 1 rue Blaise Pascal, Strasbourg F-67000, France; g.marcou@unistra.fr (G.M.); varnek@unistra.fr (A.V.); 4 Antiparasitic Chemotherapy, Faculty of Pharmacy, BioCIS, UMR 8076 CNRS-Université Paris-Sud, Rue Jean-Baptiste Clément, Chatenay-Malabry F-92290, France; sandrine.cojean@u-psud.fr (S.C.); philippe.loiseau@u-psud.fr (P.M.L.); 5 Laboratory of Microbiology, Parasitology and Hygiene (LMPH), Faculty of Pharmaceutical, Biomedical and Veterinary Sciences, University of Antwerp, Universiteitsplein 1, Antwerp B-2610, Belgium; louis.maes@ua.ac.be

**Keywords:** antimalarial, quantitative structure-activity relationships (QSAR), curcuminoid, Michael addition, *Plasmodium falciparum*, thioredoxin reductase, in silico

## Abstract

Malaria is a parasitic tropical disease that kills around 600,000 patients every year. The emergence of resistant *Plasmodium falciparum* parasites to artemisinin-based combination therapies (ACTs) represents a significant public health threat, indicating the urgent need for new effective compounds to reverse ACT resistance and cure the disease. For this, extensive curation and homogenization of experimental anti-*Plasmodium* screening data from both in-house and ChEMBL sources were conducted. As a result, a coherent strategy was established that allowed compiling coherent training sets that associate compound structures to the respective antimalarial activity measurements. Seventeen of these training sets led to the successful generation of classification models discriminating whether a compound has a significant probability to be active under the specific conditions of the antimalarial test associated with each set. These models were used in consensus prediction of the most likely active from a series of curcuminoids available in-house. Positive predictions together with a few predicted as inactive were then submitted to experimental in vitro antimalarial testing. A large majority from predicted compounds showed antimalarial activity, but not those predicted as inactive, thus experimentally validating the in silico screening approach. The herein proposed consensus machine learning approach showed its potential to reduce the cost and duration of antimalarial drug discovery.

## 1. Introduction

Malaria is a major tropical parasitic disease that each year affects 500 million humans worldwide and kills 600,000 patients, in particular children younger than five years-old and pregnant women in Sub-Saharan Africa. Although previously limited to tropical countries, the disease is now progressing in non-endemic regions as a result of both global warming and migration flows. Five parasitic species of the genus *Plasmodium*, namely *P. falciparum*, *P. vivax*, *P. ovale*, *P. malariae* and *P. knowlesi*, cause malaria, but the most prevalent and dangerous *P. falciparum* is responsible for the severe form of the disease, such as cerebral malaria.

Through the bite of a mosquito, sporozoites enter the bloodstream and infect the liver, where they divide and mature. Thousands of merozoites are subsequently produced and invade erythrocytes to initiate the intraerythrocytic cycle. The developmental cycle in the erythrocyte lasts, on average, 48 h for *P. falciparum* upon which new merozoites are released to reinvade other erythrocytes. Some of the merozoites develop into gametocytes that are taken by the mosquito during feeding, and the cycle is repeated. Digestion of host hemoglobin during the erythrocytic cycle is an important process for providing amino acids for parasitic development. The parasite detoxifies free heme as side product of hemoglobin digestion by bio-mineralization into the malarial pigment called hemozoin [1,2]. This heme detoxification is the target pathway of numerous antimalarial drugs, most of them belonging to the aminoquinoline series [3].

*Plasmodium* parasites are exposed to high fluxes of reactive oxygen species due to both the host immune response to infection and to hemoglobin digestion. To survive, they need highly active intracellular antioxidant systems providing a steady flux of glutathione (l-γ-glutamyl-l-cysteinylglycine, GSH) and reduced thioredoxin (Trx(SH)_2_). The most important antioxidative systems are based on both the glutathione reductases (GR) of the parasitized host erythrocyte and the thioredoxin reductase (TrxR) of the malarial parasite. Both flavoenzymes respectively catalyze the reduction of the disulfide bridge of oxidized glutathione (GSSG) and oxidized thioredoxin (Trx(S)_2_) into the thiol forms GSH and Trx(SH)_2_, according to Equations (1) and (2). Similar to the situation in other eukaryotes, GSH is the major low molecular mass antioxidant in malarial parasites. While classical glutathione peroxidases (Gpx) and catalase are absent, GSH plays a major role by directly reacting with hydroperoxides and by modulating enzyme activity via glutathionylation. Besides the GR and TrxR systems, *Plasmodium* has developed multiple antioxidant defense mechanisms (for reviews, see [4,5]), based on iron (Fe)-superoxide dismutase (SOD) and manganese (Mn)-SOD, as well as four unique selenoproteins, which may also represent components of the redox-regulatory network (for a review, see [6]).

(1) $$\text{GSSG}+\text{NADPH}+{\text{H}}^{+}\;\overset{GR}{\to }\;2\text{GSH}+{\text{NADP}}^{+}$$

(2) $${\text{TrxS}}_{2}+\text{NADPH}+{\text{H}}^{+}\;\overset{TrxR}{\to }\text{ Trx}{\left(\text{SH}\right)}_{2}+{\text{NADP}}^{+}$$

Both the glutaredoxin using the low-molecular weight thiol GSH and the thioredoxin pathway represent the two independent arms of the reducing system that assists the disulfide bond reduction and activation of many thioredoxin-dependent proteins [4], in particular GSSG reduction in malarial parasites. Therefore, both the glutathione and thioredoxin systems, extensively characterized in *P. falciparum*, interact closely to maintain a redox balance [7].

Hemoglobin digestion occurs in the food vacuole of *P. falciparum* and bursts into an intense liberation of reactive oxygen species (ROS), pro-oxidative heme, iron and GSSG. Heme can be degraded by GSH [8] and was shown to be inhibited by the antimalarial drugs, chloroquine and the phenolic Mannich base amodiaquine [9]. These two 4-aminoquinolines are also potent inhibitors of heme detoxification. The reaction of iron(II) with H_2_O_2_ is known to generate the highly toxic hydroxyl radical (Fenton reaction). During these processes, regulation of GSH homeostasis in *P. falciparum* depends on both GSH/GSSG efflux [10] and the de novo biosynthesis of the tripeptide [11]. Oxidation of GSH is balanced through an active GR redox cycle, while efflux is counteracted by an active GSH biosynthesis. Another central component of the antioxidative defense system of *P. falciparum* is the cytosolic thioredoxin-1 (Trx1), which directly detoxifies hydroperoxides and reduces GSSG and *S*-nitrosoglutathione (GSNO) [12]. Furthermore, Trx1 interacts with a range of proteins involved in, for example, protein folding, transcription and translation, signal transduction, glycolysis and hemoglobin catabolism [13]. TrxR has been shown to be an essential protein for the survival of *P. falciparum* blood stages [14], and therefore, a highly interesting target for specific inhibitors [15,16,17,18,19,20,21] to combat malarial parasites. 

Among the TrxR inhibitors, the natural product curcumin was reported to induce reactive oxygen species (ROS) in some cancer cells [22,23], and this is consistent with the irreversible inhibition of human TrxR in which curcumin alkylates residues (Cys496/Sec497) in the catalytic site of this enzyme [19]. However, the antimalarial activity of curcumin was shown not to correlate with *P. falciparum* TrxR inhibition [24], suggesting that the compound could kill malarial parasites by another mechanism, e.g., by ROS induction. It is known that innate immune responses against the parasite play a major role in the regulation of blood stage parasitemia through the phagocytosis of *P. falciparum* parasitized red blood cells by monocytes/macrophages. With the approach aiming at the identification of therapeutic targets to enhance this type of immunity, a key study showed that CD36 expression and CD36-mediated *P. falciparum* phagocytosis by curcumin are dependent on ROS production [25]. Because curcuminoids are known as ROS-inducing agents, these drugs can be specifically targeted for killing malarial parasites that already express high ROS levels. Therefore, enhancing innate immunity with immunomodulatory compounds like curcuminoids might contribute to the clearance of malaria parasites, including drug-resistant parasites. The drug combination of ACTs with curcuminoids will represent a valuable tool in optimizing the clearance of malaria parasites in vivo [26].

The purpose of the current study was to find new promising compounds with antimalarial activity with the aid of in silico methods that allow one to significantly accelerate the discovery of novel bioactive chemical entities. In particular, chemoinformatics relies on the existing available experimental data, allows extracting knowledge on structure-activity relationships and applies this knowledge in suggesting novel potentially bioactive compounds. In this article, we focused our efforts on the computer-aided discovery of novel antimalarial curcuminoid derivatives, with the following key steps:

(a) Collection and curation of experimental data
(b) Knowledge extraction, by construction of structure-activity relationship models
(c) Virtual screening of candidate collections and selection of candidates with best predicted properties using the above built models
(d) Experimental in vitro testing of selected candidates

In order to learn the structure-activity relationships, a maximum of reliable experimental information is required, which makes Step (a) the pivotal part of the undertaking. The particular problem in antimalarial research is not the scarcity, but the heterogeneity of the data. Potentially relevant data may cover anything from in vitro inhibitory assays on involved biological targets to functional assays using *Plasmodium* cultures. In this work, we only focused on the latter. However, screening data on *P. falciparum* can be done in very different ways, because there are various experimental conditions using different *P. falciparum* strains. An important challenge consisted of managing and fusing the various data sources: the in-house data of Davioud-Charvet’s team and literature data from the malaria subset of ChEMBL. Following this crucial step, predictive model building (b) followed a state-of-the-art protocol (see the Methods for more details), leading to a series of best models (according to statistical validation criteria) used to predict the antimalarial activity of candidate compounds, at Step (c). 

Candidate molecules were previously designed and synthetized by chemists for other applications [27,28,29,30,31]. These were both symmetrical and dissymmetrical diarylideneacetone (DAA) derivatives with variously-substituted aryl or heteroaryl rings, as well as symmetrical 2,6-diaryltetrahydrothiopyran-4-ones (2,6-DATHTPs). Experimental testing (d) of the candidates predicted to be active in silico at Step (c) led to the discovery of novel antimalarial compounds in a very cost-effective manner.

## 2. Results

Seventy two in-house compounds (35 DAA (Table S1) and 37 2,6-DATHTP (Table S2)) were virtually screened using support vector machine (SVM) classification consensus models. Out of those, thirty one candidates (19 DAAs and 12 2,6-DATHTPs) with the best predicted properties using the above built models were selected and tested in vitro for both antimalarial potencies (IC_50_: inhibitory concentration 50%) against the 3D7 *P. falciparum* strain and cytotoxicity (CC_50_: cytotoxic concentration 50%) against the human fibroblast MRC-5 cell line (Table 1).

The antimalarial activity of DAAs and 2,6-DATHTPs was assessed in cell-based in vitro cultures of the 3D7 *P. falciparum* strain (Table 1 and Table 2). Amongst in vitro tested DAAs, 16 compounds (A1–A10, A12–A13) were predicted as active, and 12 compounds (A1–A12) were observed as active. Three compounds (A11, A18, A19) were predicted to be inactive, and seven compounds (A13–A19) were found inactive against the 3D7 *P. falciparum* strain. Amongst in vitro tested 2,6-DATHTPs, eleven compounds (B1–B11) were predicted to be active, and five compounds were found as active (B1–B5). One compound was predicted to be inactive against the 3D7 *P. falciparum* strain and was indeed found as inactive (B12).

Amongst the DAAs, three compounds showed CC_50_ > 64.00 μM and were classified as not cytotoxic; six compounds (A3, A5, A9, A17 and A19) showed CC_50_ values from 29.37 μM–62.28 μM and were classified as rather not cytotoxic; eight compounds (A1 A2, A7, A10, A13, A14, A16 and A18) showed CC_50_ values from 1.06 μM–5.36 μM and were classified as rather cytotoxic (Table 1). The most cytotoxic was A18 (CC_50_ = 1.06 μM), with the same substitution pattern found on both aryl rings as found in curcumin. Amongst the 2,6-DATHTPs, five compounds (B1, B6–B8 and B10) showed CC_50_ > 64.00 μM in the assays and were classified as not cytotoxic; five compounds (B3, B5, B9, B11 and B12) showed CC_50_ values from 4.96 μM–8.00 μM and were classified as moderately cytotoxic (Table 2).

Overall, the in silico model predicted 27 compounds to be active, out of which 17 were confirmed experimentally, whereas 10 compounds did not live up to the expectations set by the prediction. Most of the correct predictions were obtained when at least one of the 17 used consensus models “voted” the compounds to be active by a large majority of >70%. The alternative selection scheme of compounds voted active by at least two models at a low majority >50% was less successful. A strong majority in consensus voting is, as already reported [33], a strong trustworthiness score of in silico predictions. All three compounds predicted as inactive were indeed found as inactive. In other words, all of the actual active one within the tested subset (17) were correctly assigned an “A” label by the in silico method, yielding a model sensitivity (or recall) of 100%. However, only three that were inactive of the actual 13 were correctly recognized as inactive; hence, the specificity score of the in silico method was only 0.23. With a balanced accuracy index of 0.62 (defined as the mean of the above-mentioned sensitivity and specificity scores) in an actual, prospective prediction challenge, the in silico model has proven effective.

## 3. Discussion

### 3.1. Known Antimalarial Curcuminoids and Unsaturated/Phenolic Mannich Bases

**Figure 1:**
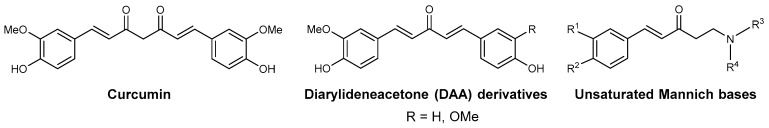
Structures of natural curcumin and diarylideneacetone (DAA) derivatives that have been identified in *Curcuma* extracts and the structure of the related unsaturated ketonic Mannich bases.

Curcumin is the major component of extracts of *Curcuma longa*, which is the base of turmeric spice. Besides its gustative and food coloring usage (food additive E100), this spice is known since antiquity for its therapeutic properties. Curcumin was reported to display a broad pattern of biological activities through pleiotropic and epigenetic mechanisms [34]. The turmeric called curcuma contains 3%–5% curcuminoids (50%–60% curcumin) and up to 5% essential oils and resins. *Curcuma longa* extracts not only contain curcumin, but also demethoxycurcumin and bisdemethoxycurcumin, as well as monocarbonyl curcuminoids, such as the “short” curcuminoid DAA analogues (R = H, or R = OMe; Figure 1) [35]. As for curcumin, DAA from *Curcuma* species are monocarbonyl curcumin analogues that were reported to display numerous biological activities [36,37,38,39], from anti-inflammatory, anticancer, antioxidant to antiparasitic activities, including antimalarial activities [24,26,40,41,42,43].

Curcumin and curcuminoids are bis(Michael acceptors) and are also related to unsaturated Mannich bases (Figure 1), which possess two electrophilic centers: (i) the α,β-unsaturated ketone is visible per se; and (ii) the other is masked and revealed only after a base-dependent deamination of the α-amino ketone. Michael addition of two nucleophiles (e.g., thiols from proteins) leads to a bis(adduct) product, upon an elimination of the dialkylamino group promoted by a base-catalyzed enolization of the ketone. From a screening of 350,000 compounds of the Pfizer library, unsaturated Mannich bases were identified to be efficient mechanism-based inhibitors of *P. falciparum* TrxR [17]. Induction of ROS by curcuminoids and related unsaturated Mannich bases has been proposed to occur via redox-cycling following Michael addition of thiols to the enone, sulfide oxidation, syn-elimination, irreversible formation of thiol overoxidation and continuous NADPH consumption [31,44].

Numerous Michael acceptors, including phenolic and C-Mannich bases, possess antimalarial potencies. First, the Mannich base pharmacophore was introduced in the 4-aminoquinolines in the 50’s of the last century, after the discovery of chloroquine, and was revealed with the discovery of amodiaquine [45] and then with tebuquine [46], amopyroquine [47] and pyronaridine [48]. These approved drugs were particularly effective at combating chloroquine-resistant malarial parasites, likely because, following phenol oxidation, these agents participated in glutathione depletion following Michael addition to the electrophilic sites of the molecule in the malarial parasites. These last effects contribute to reverse chloroquine resistance by counteracting the enhanced glutathione *P. falciparum* chloroquine resistance transporter-mediated transport in the digestive vacuole of CQ-resistant parasites [10]. Compared to amodiaquine, more metabolically-stable Mannich side chains were introduced in tebuquine (the addition of a 4-chlorophenyl group and replacement of the diethylamine by a *N*-*t*-butylamine side chain), amopyroquine (replacement of the diethylamine by a *N*-pyrrolidine) and pyronaridine (characterized by two Mannich bases’ side chains, replacement of the quinoline heterocycle by an *aza*-acridine and replacement of the diethylamine by a *N*-pyrrolidine side chain). Therefore, by delaying the Michael addition of nucleophiles to the electrophilic sites in the human host, phenolic Mannich bases behave as prodrugs of Michael acceptors. Other strategies to temporarily mask the reactive phenolic Mannich base were developed by designing Trojan horses drugs, either by tethering the phenol or the ketonic Mannich base with moieties that are recognized by a parasitic transporter to favor the drug uptake [49,50,51] or by designing hybrid molecules built from a Mannich base and a 4-aminoquinoline via an amine group [52]. The latter group was expected to be dealkylated under oxidative conditions found in malarial parasites to release two reactive entities, i.e., the Michael acceptor and the 4-aminoquinoline vector.

More recently, the alkaloids febrifugine and isofebrifugine, first found in the Chinese plant *Dichroa febrifuga* and later in the common hydrangea, have attracted considerable attention due to their potent antimalarial activity [53], opening the path for the pipeline of new antimalarial leads. The febrifugine structure of this plant-derived natural product, with its β-amino-ketone motif, is a Mannich base susceptible to Michael addition of thiols following deamination. Until now, the potential of febrifugine to react with nucleophiles under specific conditions found in malarial parasites has not been reported. As mentioned earlier, numerous acyclic Mannich bases were previously identified as *P. falciparum* TrxR inhibitors [17]. A recent review about Mannich bases identified as inhibitors of various enzymes or ligands for receptors was dedicated to their structure-activity relationships covering a broad panel of biological applications useful for medicinal chemistry and drug design [54].

### 3.2. Compound Class Selection and Organic Synthesis

Considering the numerous evidences that curcuminoids, and particularly DAAs, are potent antimalarial products, we focused our interest on this series. Both the ChEMBL database [55] and the in-house EDC database already contained this type of structure. As second category composed of 2,6-DATHP derivatives was also selected, as these may be seen as a prodrug of DAA and therefore of great interest as antimalarial candidates. Both series were recently synthesized in-house as previously described [27,28,29,30,31].

### 3.3. Virtual Screening of Candidate Collections Using the SVM Classification Consensus Models and the Selection of Candidates with the Best Predicted Properties

Most of the compounds predicted as active (Table 1 and Table 2) and also a few compounds (A11, A18, A19, B12) that were predicted as inactive against *P. falciparum* were selected for in vitro testing to validate the SVM classification consensus models. The decision on which compounds to test was based on the consensual nature of the models.

The 17 models selected for their cross-validated balanced accuracy scores above 0.7 are based on sets of various sizes—the largest spanning almost 1000 compounds, the smallest based on hardly more than 50 molecules. Note, however, that even the latter successfully withstood the quite challenging leave-1/3-out, multiply repeated cross-validation protocol. It is interesting to note that the largest set for which modelling was attempted (ChEMBL1054503, [55]), including 13,533 entries, led to a rather robust cross-validated balanced accuracy score of 0.69, making it a near miss for selection within the set of herein used models. Set size is thus not the determining factor of their modelability, and the relative success of dealing with compound sets of the above-mentioned sizes is a proof of the robustness of the modelling protocol, which was already validated in external prediction challenges [56].

Each of the 17 consensus models returns a real-value likelihood (percentage) of the candidate to qualify as active with respect to the associated property. A likelihood around 50% is inconclusive, since this means that, out of the individual distinct equations composing the consensus model, those predicting “active” and those predicting the opposite are roughly equally numerous. The closer to 100%, the higher the trust in the hypothesis that a compound will be active. Furthermore, these different “properties” are all antimalarial activity measures. It is unclear how the prediction of activity with respect to the testing protocols underlying the 17 modeled categories relates with the actual propensity of the compound under the current test conditions. The latter conditions do not completely match any of those associated with the 17 modeled properties, even though the herein used parasitic strain (3D7) and assay principle (SYBR green) are sometimes shared. For this reason, the simple rule used here was to consider a compound to be worth testing (assigned an in silico status of “A” for active by the virtual screening procedure, see Table 1 and Table 2) if at least one of the 17 predictors signaled “active” likelihood above 70%, or if more than one of the 17 predictors indicated activity likelihoods above 50%.

### 3.4. Experimental in Vitro Testing of the Selected Candidates and Structure-Activity Relationships

Finally, 41 compounds were assigned an “active” status out of the 72 submitted to virtual screening (both structural series confounded; see Table 1 and Table 2). This number was still beyond the planned biological testing effort, and therefore, only 27 of them were eventually taken. Furthermore, four additional compounds that were predicted as inactive in virtual screening were nevertheless considered for testing, because they were known as active in other in vitro parasitic tests [30,31]. Hence, this approach was an opportunity to verify their effect on plasmodial cultures, in spite of the virtual screening result. This is also very valuable for the validation of the virtual screening, as it provides the opportunity to verify whether those predicted as inactive are not “false negatives”.

A total of 31 compounds (12 DAA and 19 2,6-DATHTP: 27 of in silico status “A” vs. four “I”) were tested for both their antimalarial potencies against *P. falciparum* and cytotoxicity against human fibroblasts MRC-5 strain (Table 1 and Table 2). Within the new series of curcuminoid derivatives, novel structure-activity relationships can be evidenced, since the series covers significant chemical diversity: electron-donating and -withdrawing groups, lipophilic atoms, aromatic and polar residues, variations of the aryl/heteroaryl rings in both positions of the terminal 1,5-di(hetero)aryl penta-1,4-dien-3-one chain.

**Figure 2:**
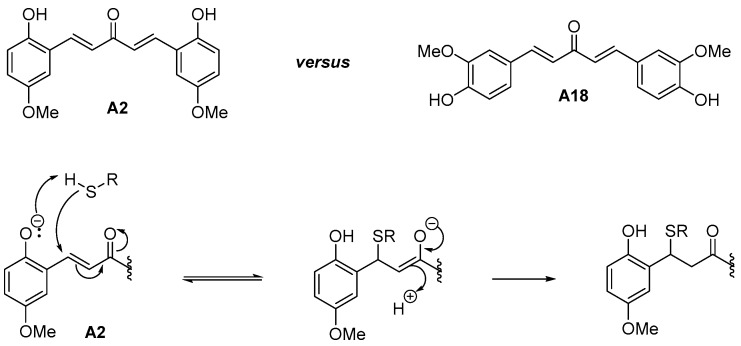
Rate enhancement of the Michael addition of thiol to the enone group of *ortho*-hydroxyl-DAA, such as the A2 molecule. This effect could account for the increased antimalarial activity observed in A2 versus A18.

As can be seen from Table 1, electron-withdrawing substituents on both aryl rings led to an increase of the antiplasmodial activity. The results showed a potent antimalarial activity for DAA bearing the same electron withdrawing group (EWG) groups (halogen atoms like Cl, F) on both aryl moieties or for heteroaryl substituted-DAA containing pyridine rings. For those most potent compounds with pyridine rings, toxicity accompanied the antimalarial potency, suggesting non-specific effects. On the contrary, the DAA bearing the same electron donating groups (EDG) groups (Me, OMe, NH_2_, NMe_2_ groups) on both aryl moieties were shown to be less active at killing parasites, with a proportional trend to display a lower cytotoxicity against human MRC-5 cells. Noteworthy, the presence of an *ortho*-hydroxyl substituent on the aromatic rings of the DAA and the associated increased antimalarial activity (IC_50_ 30.2 nM for Compound A2 vs. >500 nM for Compound A18) might be explained by the enhanced reactivity of the thiolate of the target (enzyme) thiols (resulting from lowering the pKa) through inductive/hydrogen bonding of the phenolic group (Figure 2), as previously observed [36].

Interestingly, the highest selectivity index (SI) was found for dissymmetrical DAAs with a “push-pull effect” when an EWG group was present on one aromatic ring and an EDG on the second aromatic ring of the DAA (e.g., SI values of 292.4 for Compound A5, or 625 for Compound A3, or 577.6 for Compound A6). Among all DAAs in Table 1 measured for antimalarial activity, we found discrepancies on the antimalarial potencies for four compounds in comparison with data reported previously [32]. However, as these published measurements did not show the data of positive controls (chloroquine, artemisinin), no conclusion can be drawn. Two other sets of published data [41,43] showed IC_50_ values in the sub- or low µM range close to the values found for our present series studied in this work.

With the second series of symmetrical 2,6-DATHTPs derivatives (Table 2), the aim was to mask the enone groups to decrease the toxicity against human cell lines by delaying the Michael addition of thiols from the human cells. Three types of compounds were tested in this series: sulfides, sulfoxides and sulfones. Clearly, three sulfides (B1, B2, B5) displayed slightly improved antimalarial activities with IC_50_ values in the 30–60 nM range. Both 2,6-DATHTPs with pyridyl rings are much less toxic than the corresponding DAAs. Their *S*-oxides analogues are also more active, but also more toxic, following the observed trend DAA > sulfides > sulfoxides ≈ sulfones. Worthy of mention is the remarkable activity of the DAA/*cis*-2,6-DATHTP pair with the 2,6-dichlorobenzene substitution (240.1 ± 20.4 for A12 versus 60.4 ± 10.2 for B4), but this activity did not apply for the *trans*-2,6-DATHTP B8, suggesting either a steric effect or a diastereoisomeric effect toward the target interaction. There were not enough 2,6-DATHTPs to draw general conclusions on the structure-activity relationships of this second series of compounds. However, the models allowed predicting that this series of compounds might possess antimalarial activities, and it turned out that this is the case both for the DAA series and the derived 2,6-DATHTPs.

## 4. Materials and Methods

### 4.1. Collection and Curation of the Experimental Data

**Figure 3:**
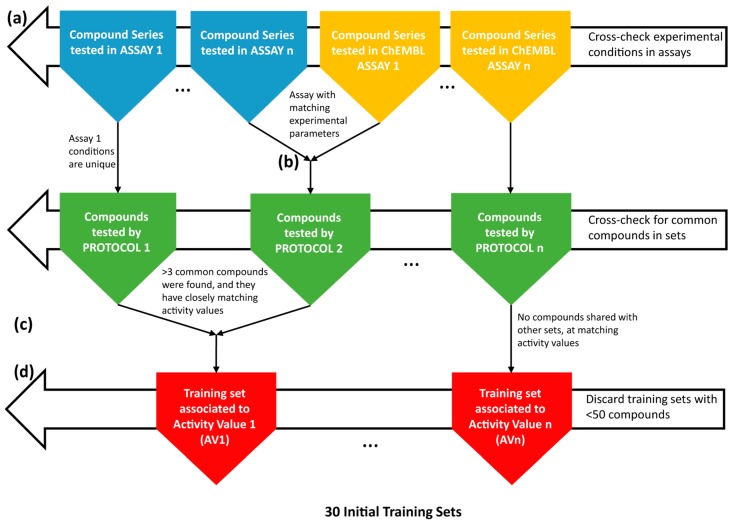
Antimalarial data merging, from various sources to QSAR model training sets. Collecting experimental series (**a**), merging of experimental series into training sets (**b**, **c**), final training sets (**d**).

Initially, eleven EDC-database (Dr. Elisabeth Davioud-Charvet’s laboratory) in-house assay-specific series, sized from 4–69 compounds, were collected in an electronic database, associating each structure to (one or several) experimentally-determined antimalarial activity values (AV) (Figure 3a). Here, AV should be understood as a generic label for antimalarial potency, expressed by different magnitudes (IC_50_, EC_50_ as the half maximal effective concentration, or ED_50_, as the median effective dose necessary to achieve 50% of the desired response, etc.) acquired under various experimental conditions (protocols). In-house data, split between different small sets associated with different activity values, are clearly reaching the critical mass needed for model building. To extend the structural diversity and number of compounds in the data series, the content of the ChEMBL database [57] was analyzed. This ChEMBL querying was targeted to specifically find compounds with antimalarial AV of the same type (IC_50_) and measured under conditions similar to the ones used for in-house series. Sixty two compound series from ChEMBL matched this query. Eventually, in order to fully exploit the malaria initiative data from ChEMBL, a second broader search was focused on the target *Plasmodium* (irrespective of the nature of the reported AV, including also EC_50_, ED_50_ values, etc.). For this, the target *Plasmodium falciparum* (Target ID: CHEMBL364), ChEMBL features 249,658 compounds with 400,176 measured AV (Figure 3a). The compounds represent 2900 different experimental assay-based series. The vast majority of these series are too small (<50 compounds) and so numerous (2870) that their in-depth analysis in view of attempted fusion cannot be pursued “by hand”, as done in this work (see the following paragraphs). The invested human effort of analysis per series would not be justified by the low number of entries (<50) per series that might be “recovered” by association to large training sets regrouping all the “compatible” data. Text mining tools should be developed for such a task, and this was beyond the scope of the present work. Herein, thirty series containing more than 50 compounds were scrutinized in terms of data quality, keeping only dose-response-based AV and discarding series with too few active compounds, or redundant series (same set of molecules, tested under nearly identical conditions with nearly identical results). Only seven series fulfilled these criteria and were used in further work.

After uploading/manual collection of in-house/ChEMBL compound series, the structural curation work, the rejection of chemically-unusable (too large, too small, too “exotic”) structures and standardization of the acceptable ones were undertaken, following the internal procedure at the basis of the virtual screening database installed on our web server powered by the ChemAxon toolkit [58]. This step specifically consists of the removal of heavy metal-containing species, of high molecular weight molecules, salts, conversion into the predicted most stable tautomer form, representation of *N*-oxides with split formal charges, conversion to the “basic” aromatic forms of 5- and 6-membered aromatic rings, etc.

After standardization, duplicates (the same structure, but different IDs) were identified and removed. Since stereochemistry is not captured by the molecular descriptors (fragment counts), training sets were reduced to stereochemistry-depleted structures. If several stereoisomers were present in a set and antimalarial activity was not affected by the stereochemical differences, then a single entry/stereoisomer was kept. If stereoisomers with widely differing AV were observed, both were eliminated. 

Merging of experimental series into training sets was performed in two steps (Figure 3b,c). First, experimental series from in-house data and from the ChEMBL database were merged if the six key experimental conditions of the protocol, as well as the nature of the reported AV were identical (Figure 3b). Merging of all of the series tested in an assay based on the same set of six key parameters (the same protocol) is a logical undertaking. In the absence of a consensus nomenclature of antimalarial tests, different groups might have run the same assay, although without referring to it by the same assay identifier. There were five compound series merged at Step 1. Ideally, this merging should have already happened within the ChEMBL malaria initiative itself and clearly shows the stringent need for the creation of a common ontology for antimalarial tests. However, even if the tests run by various groups in different locations did—as far as reported—follow the same conditions, random errors or even systematic errors due to “hidden” or unreported parameters may have arisen; hence the necessity to double-check for the coherence of the activities reported for the reference compounds.

Series emerging after Step 1 are each associated to specific experimental conditions. However, in quest of larger and more robust training sets, a second step of mergers was considered, in order to join those that contain common compounds (three, at minimum), and the reported AV values of these compounds are nearly equal (within expected experimental error, 0.5 log units for *p*IC/*p*EC measures) (Figure 3c). By this rule, 20 series (10 pairs) were fused into 10 training sets.

The machine learning strategy has been described in extensive detail [59] and can be considered as a user guide to the tool in open-access. The evolutionary SVM configurator was used here without any peculiar adaptation to this work. Interested readers will find a flowchart of the evolutionary model building scheme in Figure S1 of the Supplementary Material. Since machine learning of structure-activity relationships requires training sets to be as large and as diverse as possible, separate modelling of each protocol-specific set may still fall short of the needed critical mass of input data. This was the primary reason to assess the feasibility of fusing various protocol-specific sets into larger training sets, at Step 2. This undertaking is however not without risk. On the one hand, the fact that the activity values for the common reference compounds did not vary from one protocol to the other is, strictly speaking, only proof that the experimental parameter(s) that were changed have little impact on the activities of these specific molecules. Their lack of impact with respect to the activity of other molecules is hypothetic, and this hypothesis is all the more defendable, as the set of common reference molecules is large, with nearly constant measured activity. On the other hand, if, indeed, antimalarial screening conditions defining the protocols do have a strong impact, up to the point of witnessing an “active” compound according to Protocol A being considered “inactive” on Protocol B, then building separate models for activities measured in each protocol is, technically, the only sound alternative. However, the biological relevance of contradictory protocols must be addressed: which of Protocols A and B should, in real life, be trusted when developing an antimalarial drug? This is a non-trivial question, beyond the scope of this article. If the key protocol parameter is the choice of the *P. falciparum* strain, or its development stage, it is expectable to witness measured activities being widely different from protocol to protocol. However, differences from rather technical parameters (organism strain, drug exposure time, hematocrit %, parasitemia %, parasitic stage, assay principle, reference substances) are much more difficult to admit: do protocols differing only at such a level deserve to be considered as concurrent antimalarial activity evaluators, or should biologist first decide which choice of the “technical” parameters produces the result most relevant with respect to the actual in vivo activity? It is also highly unclear whether there is any link between the experimental setups of the antiparasitic tests and the molecular mechanisms of action involved: are there, for example, some experimental protocols better at detecting oxidative stress agents, while others preferentially highlight hemozoin formation inhibitors, etc.? The key difficulty in exploiting the antimalarial data in ChEMBL largely relies on the delicate question of the interpretation of the various testing protocols. The empirical strategy adopted in this work, fuse if common compounds seem to be coherently described by two protocols, is not perfect, but is simple and fact-based. Failure to obtain predictive models for “fused” training sets may be an indication of the failure of this fusion strategy and vice versa. Note, however, that in the absence of this fusion strategy, a significant fraction of 55 activity points of data, belonging to rather small protocol-specific sets (<50 entries) would be lost for model building.

Eventually, a number of 30 training sets, all selected because (1) each contained more than 50 molecules and (2) they span a significant activity range (i.e., contain both active and inactive), were saved in the local database (Figure 3d). Some of these sets can be directly traced back to the assay ChEMBL ID, meaning that these are single source series. They will be further on referred to by the actual assay ChEMBL ID. Sets regrouping in-house molecules with compounds from one or several ChEMBL assays considered to be based on a common protocol are fused sets and will be labelled as “FS”, followed by the protocol ID number as listed in the in-house database. Moreover, fused sets regrouping entries tested in different, but allegedly compatible protocols will be named “FS” followed by the “+”-separated concatenation of the merged protocol numbers. 

### 4.2. Knowledge Extraction by the Construction of Structure-Activity Models

Selected training sets were subjected to predictive model building. The large heterogeneity in reported AVs (IC_50_, EC_50_, etc.) and experimental protocols limited the possibility to use quantitative regression models. Therefore, the modelling strategy had to be modified to attempt categorical model building, i.e., obtaining mathematical equations that return, for any given compound encoded by its molecular descriptor, a simple “verdict”, “active” or “inactive”, rather than an estimate of the actual *p*IC_50_ or *p*EC_50_. In order to build categorical models, the real-value activity data associated with training set compounds needed first to be “binned”, i.e., converted to a binary class label (2 = active, 1 = inactive). This has been achieved for each training set by picking the best suited cut-off values separating “active” from “inactive”: on the log scale (from 7.5 to 6.0) used to report the respective activity values, cutoffs ranging from the micromolar scale were steadily increased by steps of ½ log until the “active” ones above the threshold were found to represent between 1/3 and 1/4 of the entire set.

Compounds in each set were encoded under the form of molecular descriptors. Since it is not a priori known which description strategy will be best suited to specifically capture the chemical information that best explains the antimalarial activity, a series of 39 distinct ISIDA fragment descriptor [60] sets was generated for each training set. ISIDA (for in silicon Design and data Analysis) is a collaborative project devoted to development of new methods and original software tools for structure—property modelling and computer-aided design of new compounds [60,61,62]. These 39 description schemes are the same as used in a previous work [61] and found suitable to model a large range of different biological properties. They correspond to different fragmentation strategies, using both default (by atom type), pharmacophore and force field type-based atom coloring [62].

Selecting the actual descriptor space for modelling of each set is considered as an explicit degree of freedom of the evolutionary optimizer tool [58] for the LIBSVM support vector machine algorithm [63] used here. The tool was applied to each of the 30 training sets, with default control parameters, and it produced, for each set, a family of SVM classification models by simulating the Darwinian competition of “chromosomes” encoding model building recipes (choice of descriptors out of the 39 initial options, choice of kernel type, cost parameter, etc.; see the cited work for an exhaustive discussion on the technical issues related to SVM model fine-tuning). Emerging models were ranked by a “fitness” score, which is an expression of the mean balanced accuracy of classification achieved during the repeated, three-fold cross-validation. Note that, due to the mechanism of extensive cross-validation used during the model building stage, the herein obtained predictive models are always consensus models, e.g., they are a collection of several independent predictive equations, each calibrated on random subsets of the training set and then challenged to predict the remaining items, kept out during the calibration phase. The fitness score, too, is an averaged balanced accuracy term, penalized by the standard deviation of the balanced accuracy of individual models (therefore, the actual balanced accuracy values are slightly higher than the actual fitness scores reported here, but this is not of practical relevance).

For 17 of the 30 initial sets, the top models with fitness scores exceeding 0.7 were obtained (note that a balanced accuracy level of 0.5 corresponds to random classification, whereas 1.0 stands for flawless classification of all active and inactive ones). These 17 sets are listed in Table 3, together with the thresholds used to define active and inactive classes, the number of them herewith considered active and the fitness score of the top consensus model found by the evolutionary SVM tuning tool. The experimental conditions of the one or several compatible test protocols associated with each training set can be taken from Table S3.

### 4.3. Virtual Screening of Candidate Collections and the Selection of Candidates with the Best Predicted Properties Using the Above-Built Models

Virtual screening was used to prioritize putatively active compounds within the series of novel DAAs and 2,6-DATHTPs. This series is already large enough to require a substantial biological testing budget for its exhaustive evaluation. The goal of virtual screening-based prioritization was to select the most likely active and therefore significantly reduce the screening effort.

Each of the above-built 17 SVM classification models are consensus approaches. Every predictor of each of the 17 properties is based on a family of different mathematical equations, each contributing one “vote” according to the category (active or inactive, with respect to the current properties) in which a candidate should be assigned. Therefore, the 17 consensus models return a real-value likelihood (percentage) of the candidate to qualify as active with respect to the associated property. Compounds were considered active if at least one model gave the likelihood to be active >70% or more than one model predicted the likelihood to be active >50%. If, for one property, more than 70% of the individual predictive equations forming the consensus model “agree” on labelling a compound as active, this implicitly means that the prediction is internally coherent. A low standard deviation of the predicted values stemming from individual contributors to a consensus model, which is here equivalent to “majority voting” being observed, is a strong indicator that the external molecules fall within the applicability domain of the approach [33]. In fine, using this approach, 40 compounds (26 DAAs and 14 2,6-DATHTPs) were predicted to be active and 32 compounds not to be active against the 3D7 *P. falciparum* strain (Table 1 and Table 2).

### 4.4. Experimental Testing of Selected Candidates

In vitro biological assays. Compounds were tested to establish a full dose-titration and determination of the IC_50_. The in-test concentration of DMSO did not exceed 0.5%. The selectivity antiprotozoal potential on *P. falciparum* (3D7 strain) was assessed by simultaneous evaluation of cytotoxicity on a fibroblast (MRC-5) cell line.

Antiplasmodial activity. The *P. falciparum* 3D7 strain was maintained in O+ human erythrocytes in albumin RPMI-supplemented medium under continuous culture using the candle-jar method [64]. The parasites were synchronized to the ring stage by repeated sorbitol treatment [65]. A 2.5% (*v*/*v*) erythrocyte suspension with 1% parasitemia (number of parasites per 100 red blood cells) was incubated with the compounds to be tested, previously dissolved in DMSO. Parasites were also incubated with culture medium (negative control) or with 4 µM chloroquine (positive control) in 96-well culture plates. After 44 h of incubation at 37 °C, the plates were subjected to 3 freeze-thaw cycles to achieve complete hemolysis. The useful parameter for the monitoring of in vitro susceptibility is the concentration that inhibits 50% of the parasite’s activity (IC_50_). The incorporation of SYBR Green I (Applied Biosystems) in parasite DNA was measured using the Master epRealplex cycler^®^ (Eppendorf) according to the following program to increase the SYBR Green incorporation: 90 °C (1 min), decrease in temperature from 90 °C to 10 °C (during 5 min) with reading the fluorescence. Then, the IC_50_ was calculated by icestimator software [66].

Cytotoxicity assay. MRC-5 SV2 cells were cultivated in MEM, supplemented with L-glutamine (20 mM), 16.5 mM sodium hydrogen carbonate and 5% FCS. For the assay, 104 MRC-5 cells/well were seeded onto the test plates containing the pre-diluted sample and incubated at 37 °C and 5% CO_2_ for 72 h. Cell viability was assessed fluorometrically after 4 h of the addition of resazurin. Fluorescence was measured (excitation 550 nm, emission 590 nm), and the results were expressed as % reduction in cell viability compared to untreated control. Tamoxifen is used as the reference control.

## 5. Conclusions

Extensive data curation and fusion was primordial to the extraction of large modelable structure-activity sets, in a context characterized by an extreme heterogeneity of reported antimalarial assay protocols. Noise-prone activity measures on parasite cultures are intrinsically difficult to model and are further complicated if data from various sources needs to be merged in order to reach a critical size of more than 50 compounds per set. Nevertheless, robust classification models were successfully built and applied for prospective prediction. Even though this academic virtual screening experiment is of modest scale when compared to high-throughput tests [55], it allowed successfully reducing the screening effort by a factor of two. The handpicked predicted negative added to the list of tested compounds has demonstrated the discriminating ability of the virtual screening procedure. Notwithstanding this practical success, it must be pointed out that models fitted on the basis of standard parasite culture assays cannot help in elucidating the mechanisms-of-action of the various compounds. The models only “learn” specific (sub)structural signatures associated to active and inactive and evaluate novel molecules according to learned patterns. Unfortunately, in vitro test results targeting specific parasitic targets and pathways are too sparse in public databases (such as ChEMBL) to be used for mechanism-specific model building. The success of the attempted virtual screening, in spite of lacking positive proof whether training set compounds included putative Michael electrophiles acting similarly to the alleged mechanism of curcuminoids, is encouraging: structure-activity modelling may help even in mechanistically ill-defined noisy experimental contexts.

To conclude, our work allowed predicting and identifying synthetic curcuminoids and derived 2,6-DATHTPs as potent antimalarial motifs to be developed and rationalized in QSAR studies. Interestingly, the 2,6-DATHTPs derivatives in which the enone groups were masked revealed potent antimalarial activities and lower cytotoxicity than the parent DAA. In summary, curcuminoids represent an important and under-utilized series for antimalarial chemotherapy and deserve to be developed as prodrugs, like 2,6-DATHTPs, or hybrid drugs with two reactive components, an approach that will pave the way for future development of novel, safe and effective drugs for malaria. 

## Figures and Tables

**Table 1:** Structures of diarylideneacetones (DAAs), antimalarial effects, cytotoxicity and predicted activities. 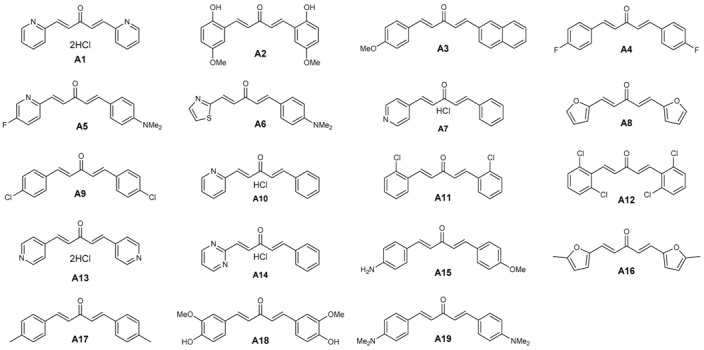

DAA Code	*Plasmodium falciparum* 3D7 Strain ^a^	Cytotoxicity MRC-5 ^b^	SI	In Silico Status *Plasmodium falciparum*	Ref.
	IC_50_ ± SD (nM)	CC_50_ (µM)	CC_50_/IC_50_	A/I	
A1	30.1 ± 8.5	1.91	63.4	A	[29,30]
A2	30.2 ± 6.3	1.88	62.2	A	[29,30]
A3	51.2 ± 10.7	32	625	A	[29,31]
A4	70.6 ± 9.7	nd	nd	A	[28,32]
A5	110.2 ± 20.3	32.22	292.4	A	[27]
A6	110.8 ± 12.5	>64.00	>577.6	A	[27]
A7	120.2 ± 17.3	1.99	16.5	A	[27]
A8	120.7 ± 10.5	>64.00	>530.2	A	[29,31]
A9	120.7 ± 12.4	42.44	351.6	A	[29,31,32]
A10	160.8 ± 20.5	1.53	9.5	A	[27]
A11	180.9 ± 29.6	29.37	162.3	A	[29,31,32]
A12	240.1 ± 20.4	>64.00	>266.5	A	[28]
A13	>500	1.13	<2.3	A	[29,30]
A14	>500	1.56	<3.1	A	[27]
A15	>500	nd	nd	A	[29,31]
A16	>500	5.36	<10.7	A	[29,31]
A17	>500	32.46	<64.9	A	[28,32]
A18	>500	1.06	<2.1	I	[29,30]
A19	>500	62.28	<124.5	I	[29,31,32]
chloroquine	20.3 ± 5.2	>64.00	>3153	A	

^a^ The standard drug chloroquine served as the positive control for the *P. falciparum* 3D7 strain. ^b^ The cytotoxicity reference drug tamoxifen exhibited cytotoxic concentration 50% (CC_50_)-values of 9.12 μM (mean of 7 values) against human MRC-5 fibroblasts. “A” means “active”, “I”, “inactive”; “SI” stands for “selectivity index” = CC_50_/IC_50_; “nd” means “not determined”.

**Table 2:** Structures of 2,6-diaryltetrahydrothiopyran-4-ones (2,6-DATHTP), antimalarial effects, cytotoxicity and predicted activities. 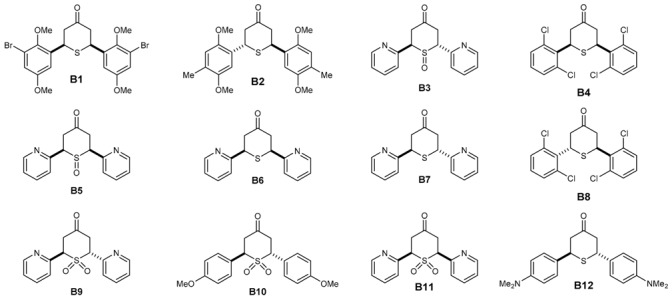

2,6-DATHTP Code	*P. falciparum* 3D7 Strain ^a^	Cytotoxicity MRC-5 ^b^	SI	In Silico Status *P. falciparum*	Ref.
	IC_50_ ± SD (nM)	CC_50_ (µM)	CC_50_/IC_50_	A/I	
B1	30.3 ± 6.1	>64.00	>2112.2	A	[28]
B2	30.6 ± 9.2	nd	nd	A	[28]
B3	40.1 ± 7.3	8	199.5	A	[29,31]
B4	60.4 ± 10.2	nd	nd	A	[28]
B5	240.1 ± 48.1	8.06	33.6	A	[29,31]
B6	>500	>64.00	<128	A	[29,31]
B7	>500	>64.00	<128	A	[29,31]
B8	>500	>64.00	<128	A	[28]
B9	>500	7.89	<15.8	A	[29,31]
B10	>500	>64.00	<128	A	[29,31]
B11	>500	7.47	<14.9	A	[29,31]
B12	>500	4.96	<9.9	I	[29,31]
chloroquine	20.3 ± 5.2	>64.00	>3158	A	

^a^ The standard drug chloroquine served as the positive control for the *P. falciparum* 3D7 strain. ^b^ The cytotoxicity reference drug tamoxifen exhibited CC_50_-values of 9.12 µM (mean of 7 values) against human MRC-5 fibroblasts. “A” means “active”, “I”, “inactive”; “SI” stands for “selectivity index” = CC_50_/IC_50_; “nd” means “not determined”.

**Table 3:** SVM classification consensus model parameters.

Training Set	Activity Threshold (log Unit)	Size	No. of Active	Cross-Validated Model Fitness Score
FS53	7.0	94	25	0.953
FS39+52	6.0	107	31	0.912
FS31	7.5	65	19	0.910
FS33+67	6.5	70	17	0.905
FS78	7.5	66	27	0.870
FS61	7.0	143	58	0.865
FS15	7.0	116	35	0.849
FS34	7.0	123	33	0.825
CHEMBL896244	7.0	225	74	0.819
FS10	7.0	59	14	0.796
FS76	7.5	160	57	0.787
CHEMBL1038869	6.5	159	43	0.730
CHEMBL730080	6.0	977	273	0.728
CHEMBL896245	7.0	113	37	0.721
CHEMBL730081	6.5	164	41	0.711
CHEMBL730641	6.5	158	51	0.702
CHEMBL1038870	6.5	156	36	0.701

## Supplementary Materials

Supplementary materials can be accessed at: http://www.mdpi.com/1420-3049/21/7/853/s1.

## Abbreviations

The following abbreviations are used in this manuscript:

ACT: artemisinin-based combination therapy
AV: activity value
DAA: diarylideneacetones
DMSO: dimethylsulfoxide
2,6-DATHTP: 2,6-diaryltetrahydrothiopyran-4-ones
GR: glutathione reductase
GSH: glutathione
GSSG: glutathione disulfide
ROS: reactive oxygen species
SI: selectivity index
SVM: support vector machine
TrxR: thioredoxin reductase
TrxS_2_: oxidized thioredoxin
