# Supplementary material for: In Silico Mining for Antimalarial Structure-Activity Knowledge and Discovery of Novel Antimalarial Curcuminoids
Source: Molecules. 2016 Jun 29;21(7):853. doi: 10.3390/molecules21070853 (PMC6273176; doi:10.3390/molecules21070853)
Supplement: Supplementary file 1 [file molecules-21-00853-s001.pdf]

Supplementary Materials: In Silico Mining for Antimalarial Structure-Activity Knowledge and Discovery of Novel Antimalarial Curcuminoids

Birgit Viira, Thibault Gendron, Don Antoine Lanfranchi, Sandrine Cojean, Dragos Horvath, Gilles Marcou, Alexandre Varnek, Louis Maes, Uko Maran, Philippe M. Loiseau and Elisabeth Davioud-Charvet

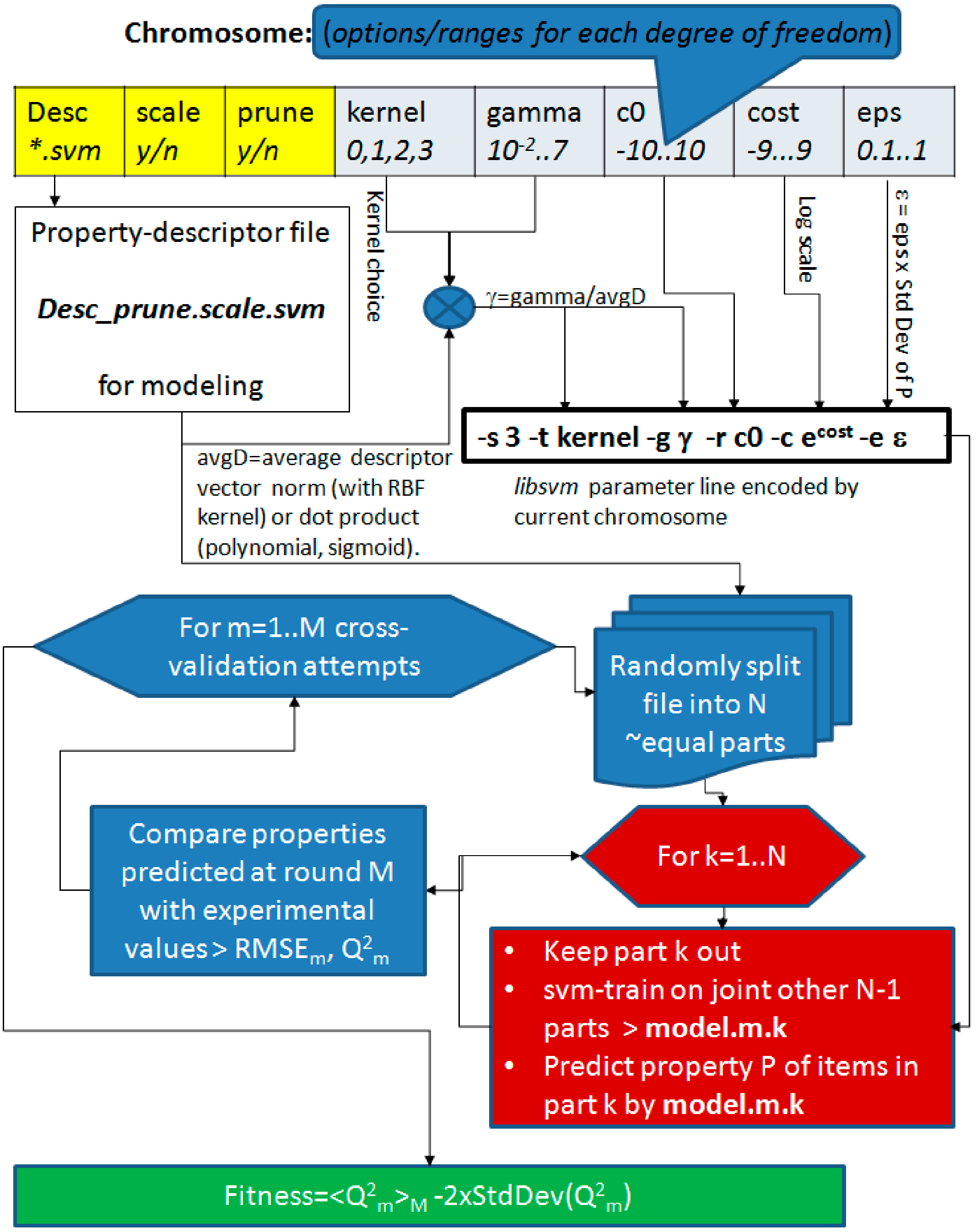

Figure S1. Schematic description of the machine learning approach.

Table S1. DAA series prediction results.

DAA Code	FS31	FS53	FS33 + 67	FS76	CHEMBL730080	*In Silico* Status
	%	%	%	%	%	A/I
A14	99.54	91.67	57.41	<50	<50	A
A16	74.54	99.07	<50	<50	<50	A
A13	<50	98.15	85.19	57.87	<50	A
A12	<50	97.22	<50	<50	54.63	A
A1	81.02	92.59	74.54	50.46	<50	A
A6	<50	91.67	<50	56.94	<50	A
A5	69.91	88.89	<50	59.72	<50	A
A7	75	82.41	51.85	<50	<50	A
A8	74.54	81.94	<50	<50	<50	A
A20	66.67	80.09	60.19	58.33	<50	A
A10	75	58.33	<50	<50	<50	A
A21	74.54	69.44	<50	<50	<50	A
A22	74.54	<50	<50	60.19	<50	A
A23	74.54	<50	<50	<50	<50	A
A24	74.54	<50	<50	<50	<50	A
A25	74.54	<50	<50	<50	<50	A
A15	74.54	<50	<50	51.85	<50	A
A26	74.54	<50	<50	<50	<50	A
A27	74.07	<50	<50	<50	<50	A
A3	73.15	<50	<50	<50	<50	A
A28	<50	<50	<50	<50	71.3	A
A4	69.91	69.44	<50	54.63	<50	A
A9	<50	69.44	<50	58.33	<50	A
A11	<50	69.44	<50	<50	<50	I
A29	<50	<50	<50	59.26	<50	I
A30	<50	<50	<50	58.8	<50	I
A31	<50	<50	<50	<50	52.31	I
A32	<50	<50	<50	<50	<50	I
A33	<50	<50	<50	<50	<50	I
A34	<50	<50	<50	<50	<50	I
A35	<50	<50	<50	<50	<50	I
A36	<50	<50	<50	<50	<50	I
A37	<50	<50	<50	<50	<50	I
A2	71.62	<50	<50	<50	<50	A
A18	<50	<50	<50	<50	<50	I
A17	71.62	68.92	<50	55.86	<50	A
A19	<50	<50	<50	<50	68.46	I

Table S2. 2,6-DATHTP series prediction results.

2,6-DATHTP Code	FS15	FS31	FS53	FS33 + 67	FS76	FS78	*In Silico* Status
	%	%	%	%	%	%	A/I
B8	<50	<50	98.15	<50	<50	54.63	A
B4	<50	<50	98.15	<50	<50	54.63	A
B7	<50	<50	93.98	77.31	<50	<50	A
B6	<50	<50	93.98	77.31	<50	<50	A
B11	<50	<50	93.06	63.89	52.31	<50	A
B10	<50	<50	90.28	77.78	<50	<50	A
B9	<50	<50	90.28	77.78	<50	<50	A
B3	<50	<50	84.72	77.78	<50	<50	A
B5	<50	<50	84.72	77.78	<50	<50	A
B13	<50	<50	83.8	<50	<50	<50	A
B14	<50	<50	83.8	<50	<50	<50	A
B15	<50	<50	67.59	<50	<50	<50	I
B16	<50	<50	67.59	<50	<50	<50	I
B17	<50	<50	67.59	<50	60.65	<50	A
B18	<50	<50	67.59	<50	<50	<50	I
B19	<50	<50	67.59	<50	<50	<50	I
B20	<50	<50	67.59	<50	<50	<50	I
B21	<50	<50	67.59	<50	60.65	<50	A
B22	<50	<50	<50	<50	60.19	<50	I
B23	<50	<50	<50	<50	59.72	<50	I
B1	<50	<50	78.38	<50	<50	<50	A
B2	<50	<50	78.38	<50	<50	<50	A
B24	<50	<50	<50	<50	<50	<50	I
B25	<50	<50	<50	<50	<50	<50	I
B26	<50	<50	<50	<50	<50	<50	I
B27	<50	<50	<50	<50	<50	<50	I
B28	<50	<50	<50	<50	<50	<50	I
B29	<50	<50	<50	<50	<50	<50	I
B30	<50	<50	<50	<50	<50	<50	I
B31	<50	<50	<50	<50	<50	<50	I
B32	<50	<50	<50	<50	<50	<50	I
B33	<50	<50	<50	<50	<50	<50	I
B34	<50	<50	<50	<50	<50	<50	I
B35	<50	<50	<50	<50	<50	<50	I
B12	<50	<50	<50	<50	<50	<50	I

Table S3. 17 protocols, their characteristics parameters and measured endpoint.

Protocol ID	Measured Property (Endpoint)	*Plasmodium* Strain	Drug Exposure Time	Hematocrit %	Parasitic Stage	Assay	Training Set	Size
10	pIC50	3D7	48 h	5.0	async	3H-hyp	FS10	65
15	pIC50	K1	72 h	2.5	async	3H-hyp	FS15	126
31	pIC50	Dd2	48 h	1.5	sync	3H-hyp	FS31	66
33	pIC50	Dd2	48 h	2.0	sync	3H-hyp	FS33 + 67	70
67	pIC50	Dd2	48 h	2.0	sync	SYBRg	FS33 + 67	70
34	pIC50	K1	48 h	2.5	async	3H-hyp	FS34	125
39	pIC50	3D7	48 h	2.0	sync	3H-hyp	FS39 + 52	120
52	pIC50	3D7	48 h	2.0	sync	SYBRg	FS39 + 52	120
53	pIC50	3D7	48 h	2.5	async	3H-hyp	FS53	94
61	pIC50	Dd2	72 h	2.0	async	SYBRg	FS61	143
76	pIC50	K1	72 h	1.25	async	3H-hyp	FS76	161
78	pIC50	K1	48 h	1.5	aync	3H-hyp	FS78	67
CHEMBL730080	pEC50	K1	72 h	2.0	sync	SYBRg	CHEMBL730080	989
CHEMBL896244	pED50	3D7	72 h	0.5	async	3H-hyp	CHEMBL896244	230
CHEMBL896245	pED50	K1	72 h	0.5	async	3H-hyp	CHEMBL896245	201
CHEMBL1038869	pEC50	SB-A6	72 h	2.0	sync	SYBRg	CHEMBL1038869	163
CHEMBL1038870	pEC50	D10	72 h	2.0	sync	SYBRg	CHEMBL1038870	160
CHEMBL730081	pEC50	3D7	72 h	2.0	sync	SYBRg	CHEMBL730081	168
CHEMBL730641	pEC50	K1	72 h	2.0	sync	SYBRg	CHEMBL730641	162
